# Conservatively Treated Mesenteric Vein Thrombosis in a 48-Year-Old Obese Female: A Case Report

**DOI:** 10.7759/cureus.49966

**Published:** 2023-12-05

**Authors:** Rita Fernandes, Estefania Curralo, Silvia Cunha, Fabíola Ferreira

**Affiliations:** 1 General Practice, Unidade Local de Saúde (ULS) do Alto Minho, Viana do Castelo, PRT; 2 Family Medicine, Unidade Local de Saúde (ULS) do Alto Minho, Viana do Castelo, PRT

**Keywords:** anticoagulants, case reports, obesity, conservative treatment, mesenteric ischemia

## Abstract

Mesenteric vein thrombosis (MVT) is a rare pathological entity that results in compromised venous return from the intestine due to involvement, in most cases, of the superior mesenteric vein. Its diagnosis is not straightforward, since the findings on physical examination are often disproportionate to the patient's pain complaints, leading to it being undervalued by clinicians.

The patient is a 48-year-old female with a medical history of essential arterial hypertension, dyslipidemia, class II obesity, and Hashimoto's thyroiditis. She also had a family history of gastric and colon cancer, with an age at diagnosis of over 70 years. She went to an appointment at a primary care facility for abdominal pain located in the left hypochondrium and flank, with ipsilateral lumbar irradiation and no other accompanying symptoms. Physical examination revealed a globose, depressible abdomen, painful on palpation of the left quadrants, with no other associated signs of peritoneal irritation. Due to suspicion of acute diverticulitis, the patient was referred to the emergency department (ED) for assessment by general surgery. In the emergency department, given the patient's body type and the fact that the physical examination findings were disproportionate to her symptoms, an abdominal and pelvic computed tomography (CT) scan was ordered, which revealed complete thrombosis of the entire length of the inferior mesenteric vein, with a focal extension of the thrombus, partially obstructing the confluence with the superior mesenteric and portal veins. Various complementary diagnostic tests were requested, which revealed no clinically significant findings, and obesity was therefore identified as the only risk factor. In this context, the patient started anticoagulation with warfarin, with the indication that it should be ad aeternum. To date, the patient remains asymptomatic, and there have been no new thrombotic events.

Given the high morbidity and mortality rates of this pathological entity, it is imperative that clinicians are trained to recognize the typical signs of mesenteric venous thrombosis, in the characteristic epidemiological context, in order to establish a timely diagnosis and carry out early targeted therapeutic intervention.

## Introduction

Mesenteric vein thrombosis (MVT), responsible for 5%-15% of all mesenteric ischemic events, is a rare pathological entity that is difficult to diagnose and preferentially affects the superior mesenteric vein [1,2]. The impairment of venous return from the intestine, associated with a delay in detection and, consequently, in the institution of anticoagulant therapy, can lead to the occurrence of mesenteric infarction, which is the most serious and immediate complication of MVT and is associated with a mortality rate of 60% [3]. Early initiation of anticoagulant therapy is associated with a very low incidence of this complication, since it aims to prevent the thrombus from extending and, at the same time, to ensure reperfusion of the affected vessel [4,5]. Despite being a relatively rare condition with a low incidence, the diagnosis of MVT has increased over the last two decades since the advent of computed tomography (CT), since it identifies around 90% of cases early [4-8]. Even so, mortality remains high due to its presentation with non-specific symptoms, the delay in diagnosis, and the low level of suspicion due to a lack of awareness among clinicians [2,9].

The diagnosis of MVT is difficult, as the clinical manifestations are non-specific and often disproportionate to the patient's pain complaints, leading to doctors not considering it as a differential diagnosis [3]. So, the presentation of this clinical case aims to draw attention to the occurrence of this rare pathology, which is particularly relevant, as it involves thrombosis of the more rarely affected inferior mesenteric vein.

## Case presentation

Our patient was a 48-year-old female, a doctor, with a medical history of essential arterial hypertension, dyslipidemia, class II obesity (body mass index (BMI): 35.8 kg/m^2^), and Hashimoto's thyroiditis and a surgical history of two caesarean sections (2004 and 2005). She also had a family history of gastric cancer (mother and three first-degree maternal uncles) and colon cancer (one first-degree maternal uncle) with an age at diagnosis of over 70 years. She was medicated with atorvastatin 20 mg + perindopril 5 mg + amlodipine 5 mg, levothyroxine sodium 0.137 mg (all once daily), and etonogestrel 11.7 mg + ethinylestradiol 2.7 mg (vaginal ring). She reported full compliance. No drug allergies were registered.

The patient attended an appointment at a primary care facility for abdominal pain located in the left hypochondrium and flank with ipsilateral lumbar irradiation, which had been going on for two days and was rated 8/10 on the numerical pain intensity scale. She identified walking as an aggravating factor, even adopting an antalgic position with convexity to the right. She mentioned the need for alternating doses of acetaminophen 1 g and ibuprofen 400 mg without complete resolution of the pain complaints. She reported nausea without vomiting. She denied changes in gastrointestinal transit, urinary complaints, fever, or any other accompanying symptoms.

Physical examination revealed a globose, depressible abdomen that was painful on palpation of the left quadrants, especially in the left iliac fossa. There were no other signs of peritoneal irritation. There were no other significant findings on physical examination. On suspicion of acute diverticulitis, the patient was referred to the emergency department (ED).

**Figure 1:**
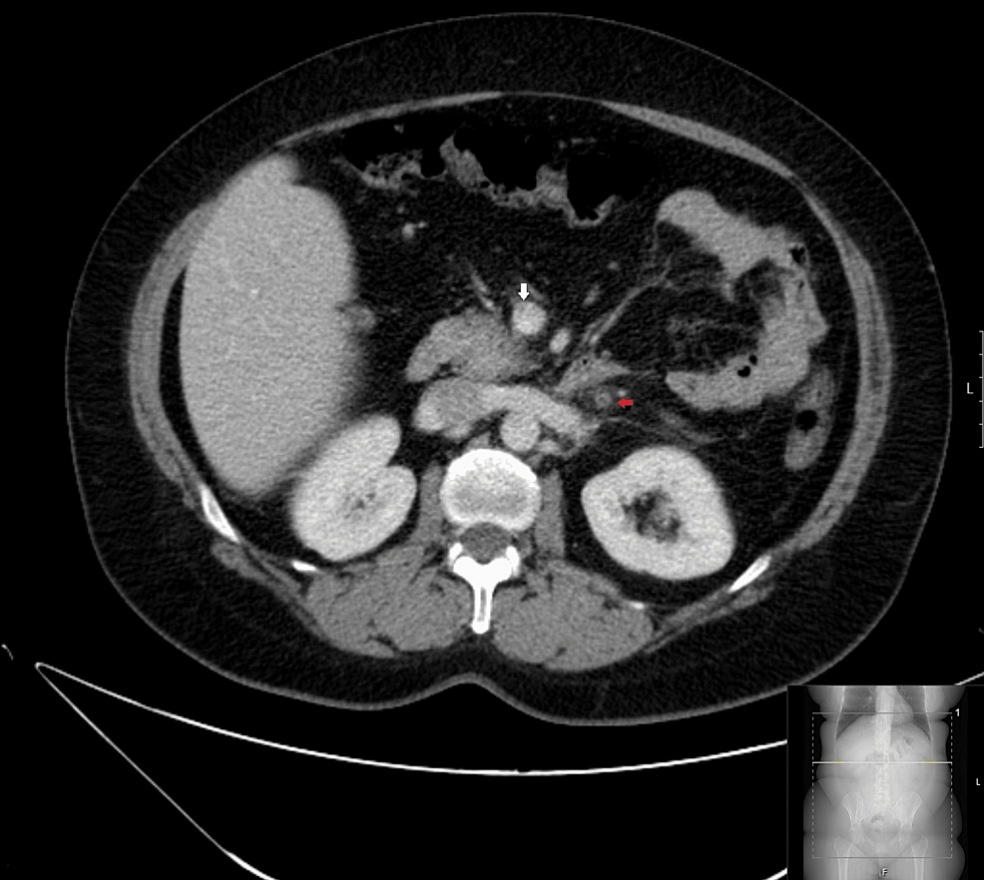
CT with axial reformatting after intravenous contrast administration (portal phase). The thrombosed inferior mesenteric vein is identified, without endoluminal contrast (red arrow). Note the normal contrast filling of the superior mesenteric vein (white arrow). CT: computed tomography

**Figure 2:**
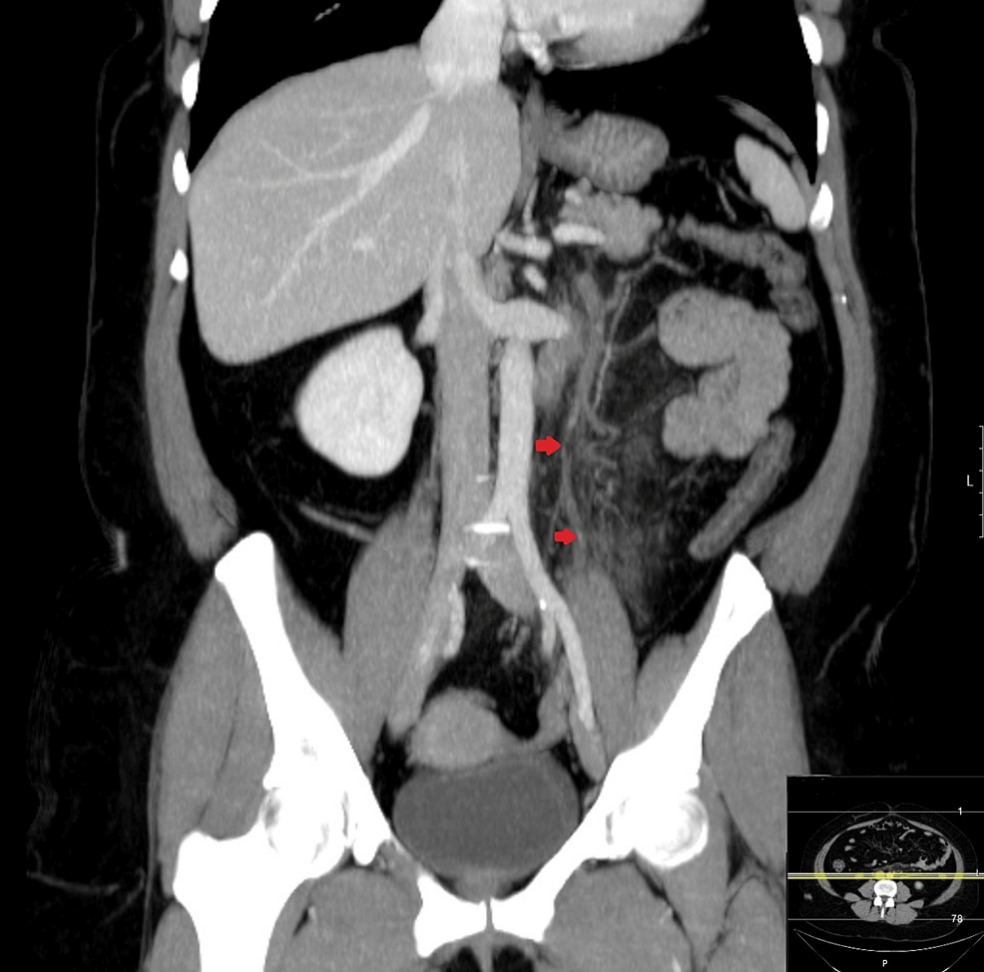
CT with coronal MIP reconstruction after the administration of intravenous iodinated contrast (portal phase). There is extensive thrombosis of the inferior mesenteric vein and its tributaries, associated with surrounding mesenteric edema (red arrows). CT: computed tomography, MIP: maximum intensity projection

**Figure 3:**
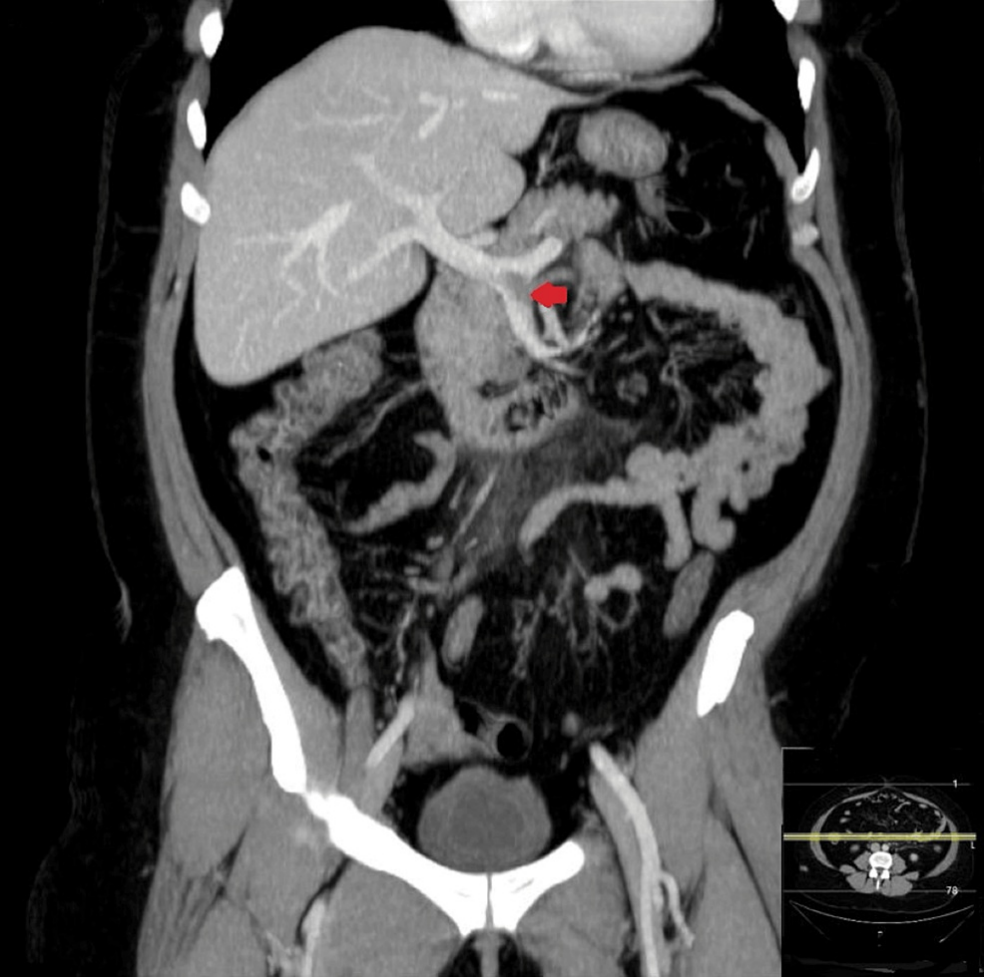
CT with coronal MIP reconstruction after the administration of intravenous iodinated contrast (portal phase). Thrombosis of the inferior mesenteric vein up to its confluence with the proximal superior mesenteric vein (anatomical variant), continuing with a partial thrombus extending to the spleno-portal confluence (red arrow). CT: computed tomography, MIP: maximum intensity projection

In the ED, given the patient's body type and the fact that the physical examination findings were disproportionate to her symptoms, an abdominal and pelvic computed tomography (CT) scan was requested, the report of which revealed "complete thrombosis of the entire length of the inferior mesenteric vein and tributaries to the rectosigmoid segment, accompanied by significant densification of the fat planes surrounding these venous segments, suggesting edema, including thickening of the anterior renal fascia and left periovarian planes. There is a focal extension of the thrombus partially obstructing the confluence with the superior mesenteric and portal veins." Figure 1 shows an axial section from this scan, while Figure 2 and Figure 3 show two coronal sections. Thus, due to extensive mesenteric venous thrombosis, the patient was admitted to the hospital, under the care of the internal medicine department.

Once the diagnosis was established, she was started on anticoagulation with enoxaparin at a dose of 1 mg/kg every 12 hours, with no record of associated blood loss. At the same time, various complementary diagnostic methods were requested, with the aim of investigating the etiology of the aforementioned thrombotic event. Thus, anticardiolipin antibodies, anti-beta-2-glycoprotein I, and lupus anticoagulant were requested, with negative results; there was no factor V Leiden mutation, *G20210A* in the prothrombin gene, and *JAK-2 V617F* or calreticulin gene mutation; the assay for protein C, S, and antithrombin activity showed no changes; there was no resistance to activated protein C; the homocysteine assay showed no changes. Still in the hospital and considering the family history of cancer of the patient, she underwent upper and lower digestive endoscopy, which did not reveal any changes. She also underwent a positron emission tomography (PET) scan, which showed no significant findings. There were no complications during her hospital stay, and she was asymptomatic on discharge.

With regard to the proposed monitoring and treatment, the patient was kept on anticoagulation, levothyroxine sodium, and antihypertensives under the previously mentioned dosage regimen until she was re-evaluated on the next outpatient appointment. In addition, given her low-density lipoprotein (LDL) value (197 mg/dL) on admission to the ED, in association with the thrombotic event that had occurred, she was started on a high-potency statin (rosuvastatin 20 mg daily) instead of atorvastatin 20 mg. The vaginal ring (etonogestrel 11.7 mg + ethinylestradiol 2.7 mg) was discontinued, even though there is only evidence that combined oral contraceptives are a risk factor for thrombotic events.

About a month later, the patient attended the internal medicine appointment and started anticoagulation with warfarin, aiming for an international normalized ratio (INR) between 2 and 3. At the six-month follow-up appointment, since the patient was unsuccessful in reducing her BMI, the warfarin regimen was made permanent. To date, the patient reports compliance and denies the occurrence of any adverse effects. She continues to be followed up at her primary care center for anticoagulated patients in order to monitor her INR and is monitored at the internal medicine outpatient appointment at the hospital every six months. She remains asymptomatic, and there have been no new thrombotic events. As the patient has no indication to remain under permanent anticoagulation, this issue will be reviewed at the next appointment.

## Discussion

Primary MVT, which accounts for around half of all cases, is considered spontaneous and idiopathic when there is no identified etiology or predisposing factor [10]. However, this figure has been decreasing with the increase in the index of suspicion and with the improved diagnosis of coagulation disorders [10]. On the other hand, secondary MVT results from an underlying disease or associated risk factor, and primary prothrombotic states are its most common causes. Prothrombotic states such as heparin-induced thrombocytopenia, essential thrombocytosis, post-splenectomy thrombocytosis, polycythemia vera, and neoplastic diseases are identified in 60%-75% of patients with MVT [10]. Genetic abnormalities also contribute to venous thrombosis and primary hypercoagulable states. These include defects in antithrombotic proteins (antithrombin III, protein C, and protein S) and increased prothrombotic proteins (resistance to activated protein C and mutation of the prothrombin gene *G20210A*) [2,9,11]. Combined mesenteric and portal venous thromboses are more often associated with non-systemic pathologies, such as local abdominal inflammatory diseases (inflammatory bowel disease, pancreatitis, and diverticulitis), myeloproliferative neoplasms, and other malignant diseases (hepatocellular carcinoma and pancreatic adenocarcinoma) [10].

In dealing with this patient, it was found that the causes associated with secondary MVT were ruled out by requesting the aforementioned complementary diagnostic tests, which was a positive aspect in the management of this case. Thus, MVT is assumed to occur in the context of obesity (class II), which, in itself, is a risk factor for events of this nature [3]. In addition to clinical factors such as immobility, obstructive sleep apnea syndrome, heart failure, and venous stasis, the main proposed mechanisms responsible for obesity-associated thrombosis are impaired fibrinolysis and chronic inflammation [12]. Adipokines and pro-inflammatory cytokines secreted by M1 macrophages within adipose tissue contribute to the upregulation of procoagulant factors such as tissue factor and plasminogen activator inhibitor 1 (PAI-1), resulting in increased thrombin production, greater platelet activation, reduced fibrinolysis, and an increased risk of thrombosis [3]. The effect of obesity on venous thrombosis can be even more pronounced in portal vein thrombosis, which can spread to the splenic vein and/or mesenteric veins [3,13]. In fact, concentrations of inflammatory molecules known to activate endothelial cells, making them more pro-thrombotic, including interleukin 6, are higher in the portal vein than in the radial artery of obese patients [3,14,15]. A patient with an acute mesenteric vein thrombosis presents with a sudden clinical picture of non-specific symptoms that usually include abdominal pain, nausea, and vomiting, with pain being the predominant symptom [16]. Given that the clinical complaints were disproportionate to the findings on physical examination, a CT scan was essential in this patient's diagnosis. However, the value of an ultrasound should not be underestimated, as it provides a high sensitivity and specificity at a lower cost and without the use of radiation [16]. Conversely, D-dimers are non-specific and may be elevated as a result of another abdominal process. Therefore, they cannot currently be recommended as a first-line diagnostic tool [17].

In the absence of treatment, the abdominal pain will worsen with the progressive onset of peritonitis, possibly culminating in intestinal infarction. Anticoagulation is the first-line therapy for patients with acute symptomatic MVT, providing benefits that include preventing intestinal ischemia, reducing hospital stays, and increasing survival [9,16]. It should be noted that anticoagulation was administered to this patient as soon as the diagnosis was established, thus adding a positive aspect to the approach in this case. The American College of Gastroenterology suggests at least six months of anticoagulation in patients with MVT without demonstrable thrombophilia and when the etiology of the thrombosis is reversible, and anticoagulation for an indefinite period in patients with MVT and associated thrombophilia [16].

## Conclusions

Understanding mesenteric venous thrombosis allows us to recognize suggestive epidemiological pictures and contexts, supporting the clinical suspicion that will lead to diagnosis. Timely diagnosis and an appropriate therapeutic approach are factors that influence prognosis and can potentially change the current paradigm of the disease, helping to reduce high rates of morbidity and mortality. It is, therefore, the responsibility of all clinicians to recognize this pathology at an early stage. However, in addition to exploring the disease, the primary care physician must also manage the pain. Therefore, helping to manage the impact that these clinical situations have on the patient's life, both professionally and personally, by getting to know their feelings, ideas, expectations, and fears, is a must for clinical practice as a family doctor.
